# A Study of Comparative Advantage and Intra-Industry Trade in the Pharmaceutical Industry of Iran

**DOI:** 10.5539/gjhs.v7n6p295

**Published:** 2015-04-23

**Authors:** Hassan Yusefzadeh, Aziz Rezapour, Farhad Lotfi, Farbod Ebadifard Azar, Bahram Nabilo, Hassan Abolghasem Gorji, Mohammad Hadian, Niusha Shahidisadeghi, Atiyeh Karami

**Affiliations:** 1 Department of Public Health, School of Health, Urmia University of Medical Sciences, Urmia, Iran; 2 Department of Health Economics, School of Health Management and Information Sciences, Health Management and Economics Research Center, Iran University of Medical Sciences, Tehran, Iran; 3 Department of Health Economics, School of Management and Medical Information, Shiraz University of Medical Sciences, Shiraz, Iran; 4 Department of Public Health, school of Health, Iran University of Medical Sciences, Tehran, Iran; 5 Department of Health Services Management, School of Health Management and Information Sciences, Iran University of Medical Sciences, Tehran, Iran; 6 Health Management and Economics Research Center, Iran University of Medical Sciences, Tehran, Iran

**Keywords:** pharmaceutical products, comparative advantage, intra-industry trade, trade specialization

## Abstract

**Background:**

Drug costs in Iran accounts for about 30% of the total health care expenditure. Moreover, pharmaceutical business lies among the world’s greatest businesses. The aim of this study was to analyze Iran’s comparative advantage and intra-industry trade in pharmaceuticals so that suitable policies can be developed and implemented in order to boost Iran’s trade in this field.

**Methods:**

To identify Iran’s comparative advantage in pharmaceuticals, trade specialization, export propensity, import penetration and Balassa and Vollrath indexes were calculated and the results were compared with other pharmaceutical exporting countries. The extent and growth of Iran’s intra-industry trade in pharmaceuticals were measured and evaluated using the Grubel-Lloyd and Menon-Dixon indexes. The required data was obtained from Iran’s Customs Administration, Iran’s pharmaceutical Statistics, World Bank and International Trade Center.

**Results:**

The results showed that among pharmaceutical exporting countries, Iran has a high level of comparative disadvantage in pharmaceutical products because it holds a small share in world’s total pharmaceutical exports. Also, the low extent of bilateral intra-industry trade between Iran and its trading partners in pharmaceuticals shows the trading model of Iran’s pharmaceutical industry is mostly inter-industry trade rather than intra-industry trade. In addition, the growth of Iran’s intra-industry trade in pharmaceuticals is due to its shares of imports from pharmaceutical exporting countries to Iran and exports from Iran to its neighboring countries.

**Conclusions:**

The results of the analysis can play a valuable role in helping pharmaceutical companies and policy makers to boost pharmaceutical trade.

## 1. Introduction

The pharmaceutical industry should be examined from two perspectives; economic and social. Drugs have a strategic nature, are essential, vital and stabilizing and are considered as one of the few strategic commodities. As a result, they are equal to food and security regarding their importance and significance. Drug is a key element of the health care system. More than 75% of medical treatments involve drugs. Many of preventative health care services are dependent on drugs. Drug accessibility is an indicator of the quality of health care services. Thus, drug inaccessibility will have widespread welfare and security consequences for countries (Shabaninejad, Mehralian, Rashidian, Baratimarnani & Rasekh, 2014). The drug is also remarkably important and valuable from the economic perspective. Regarding the economic value, pharmaceutical industry is one of the most capital-intensive, profitable and vital industries in the world (Cheraghali, 2010).

Pharmaceutical trade is one of the largest businesses in the world. Large pharmaceutical companies spend a lot of money to produce new drugs and consequently increase their assets (Barouni, Ghaderi, & Banouei, 2012). Studies on the status of drug market indicate a global progressive increase. The rate of drug sales around world has increased from 395 billion dollars in 1998 to 954 billion dollars in 2012 (approximately 2.4 times more) (International Trade Center, 2012). The global drug sales rate is expected to reach to 1.3 trillion dollars until 2020. Thus, the growth rate of other industries is very low compared with the growth rate of global pharmaceutical market (Davari, Walley, & Haycox, 2011).

Drug costs and expenses associated with drug consumption in Iran and many developing countries are about 30% of the total health care expenditure and approximately 50% of outpatient health and medical care expenses, but this share in most OECD countries is less than 10% (Bidel & Camousi, 2009). Pharmaceutical industry continues to develop and construct a significant part of international trade until recognition of exist disease treat (Organization for Economic Co-operation and Development (OECD): the Pharmaceutical Industry, 2012).

Entry to international drug markets requires the development of this advanced industry. The development of this knowledge-based industry, in turn, requires research and development (R&D). These R&D programs have some spillover effects. The effects expand other economic sectors by producing new products (often resulting in higher productivity) and generate employment (Boring, 2010). Because this industry is determined by a wide range of economies of scale and differentiated products, it is expected that international trade in this industry engages intra-industry trade (Nasiri & Haji Hassani, 2013).

Iran’s share of the pharmaceutical industry is about 2.6 billion dollars (out of the total 1,200 billion dollars). This has hardly helped promote Iran’s intra-industry trade in the recent years (Pharmaceutical Statistics, 2012).

With respect to economic integration, trade liberalization probably has considerable adjustment costs in Iran’s pharmaceutical sector which can be reduced by increasing intra-industry trade (IIT) in this sector through eliminating barriers of bilateral trade, creating trade zones and customs unions, as well as taking care of existing comparative advantages. Thus, the extent of IIT and total trade among Iran and these countries will increase (Tayebi & Azerbaijani, 2009).

Furthermore, regarding the importance of Iran’s IIT in pharmaceuticals in non oil exports, this sector can help in absorbing foreign exchange (Kebriaeezadeh, Koopaei, Abdollahiasl, Nikfar & Mohamadi, 2013). Also, Iran’s relative advantages in pharmaceutical producing comprise a plenty of natural resources, relative specialty in production and exports of pharmaceuticals, skilled labor and the greater proximity to Islamic countries’ markets that these advantages accentuate this industry (Basmanjy, Izadi, & Behbahani, 2008).

Due to the importance of drug and its high expenses, the rapid growth of Iran’s pharmaceutical industry and manufacturing capabilities, the substantial role of drugs in patients’ medical treatment process and generally society’s health, annual departure of large amounts of foreign exchange in order to import drugs from country, the high value added found in Iran’s pharmaceutical industry, and government’s policies to support domestic production and promote exports, it is necessary to assess and analyze the comparative advantage and intra-industry trade in pharmaceutical industry in order to identify Iran’s competitive potentials, design and implement adequate policies to boost pharmaceutical trade, and promote pharmaceutical exports. The results can be used in foreign trade planning as a strategic and decision-making tool.

The aim of research is to calculate and evaluate Iran’s comparative advantages and intra-industry trade in pharmaceuticals. Finally, some constructive recommendations are made to promote exports of pharmaceuticals.

No study associated with comparative advantage and IIT of Iranian pharmaceutical industry was found. Though, there are many studies related to different economic sectors some of which would be cited.

Rasekhi (2008) assessed intra-industry trade of Iran’s agricultural products via the Grubel-Lioyd and Fontagn-Freudenberg indexes in the 1997-2003 periods. The Results showed low but increasing IIT for agricultural products of Iran. Specifically, the IIT of Iran’s agricultural products is estimated about 2.73-5.98 percent during the time period. Therefore, it seems that foreign trade in agricultural products of Iran is based on comparative advantage currently (Rasekhi, 2008).

The other study associated with the comparative advantage has been carried by Mehdipour et al. (2006) for potato production in Iran in the 1999-2001 years. The results of Policy Analysis Matrix (PAM) indicated that Iran has a comparative advantage in potato production (DRC=0.54), nominal protection coefficient of product showed the indirect tax on potato production (NPC= 0.49), nominal protection coefficient of input indicated the indirect subsidy on input (NPI = 0.54) and effective protection coefficient indicated that the indirect tax is more than the subsidy that paid for input by government. In other words, the government policy for paying subsidy on inputs should be changed to effective system (Mehdipor, Sadrolashrafi, & Kazemnejad, 2006).

Savin and Winker (2009) calculated Russian revealed comparative advantages over the 2004 to 2009, and demonstrated that the Russian Federation has prospective advantages in some medium and high technological industries like pharmaceutical industry, electronic equipment, machinery building and railway transport as well as in some other industries like production of clothes. Also, they proposed to understand these prospective advantages, a policy with precise motivating tools needs to be used to these industries (Savin & Winker, 2009).

## 2. Methods

### 2.1 Comparative Advantage: Definition and Indicators

Generally and practically, comparative advantage can be defined as follows: the ability and authority of a country to manufacture and export cheaper and higher quality commodities or the ability to produce cheaper and higher quality commodities in the target markets to other countries. In the above definition, the comparative advantage refers to a country’s ability to manufacture and export less costly goods and products that are relatively cheaper than the other similar goods manufactured and exported by other countries and foreign competitors. In other words, under the same conditions, a country can benefit from its comparative advantage that is able to introduce cheaper and higher quality goods into the target markets and deliver cheaper products with high quality to the consumers (Salvatore, 2012).

The main indicators were used in this research for examine Iran’s comparative advantage in pharmaceuticals include the measures of trade specialization, export propensity, import penetration, revealed comparative advantage and revealed competitive advantage indexes, that are explained as following.

A) Equation of trade specialization index to measure Iran’s comparative advantage in pharmaceuticals is as follows:


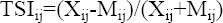


Where, X_ij_ and M_ij_ are respectively exports and imports of good i by country j. The value of trade specialization index lies between -1 and +1. The positive value of TSI shows that the country has specialization in produce of good i, and is a net exporter of good i. So, the country appears to have a comparative advantage in trade of that good. Conversely, the country seems to have a comparative disadvantage in trade of good i if the value of TSI is negative, and is a net importer of good i (Pourmoghim, 2011).

B) Export propensity index for Iranian pharmaceutical industry is calculated as the percentage ratio of Iran’s pharmaceutical exports over its domestic production (Asgharzadeh, 2009). That is:


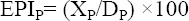


Where, X_P_ and D_P_ are exports and total domestic production of pharmaceuticals in Iran, respectively.

Total domestic production is measured as follows:


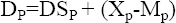


Where, DS_P_ and M_p_ are respectively total domestic sales and imports of pharmaceuticals by Iran.

C) Import penetration ratio, which is an indicator of international competition, for Iranian pharmaceuticals industry is measured as the percentage ratio of pharmaceutical imports over total domestic sales or clear consumption of pharmaceuticals in Iran (Gerald, 2009). That is:


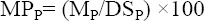


D) Exports to imports ratio are calculated to determine the competitiveness in Iran’s pharmaceutical trade.

E) Revealed comparative advantage index of Balassa mention to ratio of a country’s export share over world export share for a special good. That is:


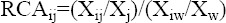


Where, RCA_ij_, X_ij_, X_j_, X_iw_ and X_w_ are Balassa’s index of revealed comparative advantage, exports of good i by country j, exports of all goods by country j, exports of good i by all countries in the world and exports of all goods by all countries in the world, respectively.

If the value of index is less than one, the country has a revealed comparative disadvantage (loss) in the trade of good i and reversely, if the ratio is greater than one, the country has a revealed comparative advantage in the trade of that good. The trend of RCA_ij_ shows the changes in export share of commodity i to country j and its large fluctuations can be a clear proof of absence an apparent strategy for export of that good (Balassa, 1965).

f) Vollrath’s measures are used to examine revealed competitive advantage in pharmaceuticals for Iran and other exporting countries, which calculates revealed competitive advantage in terms of three measurements: Relative Trade Advantage (RTA), Relative Export Advantage (REA) and Revealed Competitiveness (RC), as follows:


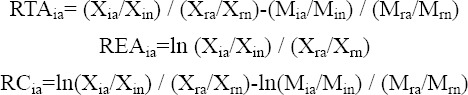


Where, RTA_ia_, REA_ia_ and RC_ia_ are relative trade advantage, relative export advantage, revealed competitiveness of country i in good a, respectively. X_ia_, M_ia_, X_in_, M_in_, X_ra_, M_ra_, X_rn,_ M_rn_, r and n are respectively exports and imports of good a by country i, exports and imports of all goods except for good a by country i, exports and imports of good a by all countries in the world excluding country i, exports and imports of all goods excluding good a by all countries in the world except for country i, world minus country i and all traded goods minus good a.

RTA and RC comprise both export and import parts which are more compatible with the real world trade. The positive values of RTA, REA or RC represent a competitive advantage, whereas the negative value indicates a competitive disadvantage (Vollrath, 1991).

### 2.2 Theory of Intra-Industry Trade and Measurement

Intra-industry trade is defined as the simultaneous export and import of goods of the same industry between two trade partners. Intra-industry trade that engages exports and imports within the same industry has become more important in international trade in recent decades. Not only developed countries but also developing countries have acquired great profits from such trade. The trade of same product groups is due to customer demand for different preferences between trading partners. These products are not alike but are differentiated by design, style and functional characteristics. Therefore, goods are differentiated by their quality (that is called the vertical IIT) or appearance (that is named the horizontal IIT) (Sawyer, Sprinkle, & Tochkov, 2010).

This phenomenon cannot be essentially described by traditional theories, may be explained and calculated by IIT models. Because of the rising interest in IIT and the important trading relationship with neighboring countries, it is necessary to investigate IIT between Iran and trading partners. A considerable IIT may have also significant policy implications (Blanes & Martín, 2000).

Recognition of comparative advantages is of greatest importance, when we are looking for an appropriate model for producing, export and import of products in order to increase our foreign relationships. Intra-industry trade facilitates countries discover the actual comparative advantages of their goods by comparing themselves with other countries in relation to technologies and production methods (Gullstrand, 2002).

In this study, we compute the extent of Iran’s intra-industry trade in pharmaceuticals by using the Grubel–Lloyd index (1975). This index is defined as:


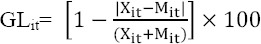


Where X_it_ and M_it_ indicate the values of export and import of industry i in year t, respectively. The intra-industry trade index varies from 0 (complete inter-industry trade) to 100 (complete intra-industry trade) (Wei, 2004).

Using of Grubel-Lloyd index in clarifying the growth of intra-industry trade over time leads to misleading and biased results. Therefore, the developed indexes of Menon-Dixon are used to measure changes in intra-industry trade in terms of the shares of exports and imports to growth in total trade (TT_i_), net trade (NT_i_) and IIT (IIT_i_).


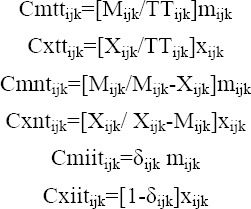


Where, Cmtt_ijk_, Cxtt_ijk_, Cmnt_ijk_, Cxnt_ijk_, Cmiit_ijk_ and Cxiit_ijk_ are respectively shares of import growth to growth in total trade, shares of export growth to growth in total trade, shares of import growth to growth in net trade, shares of export growth to growth in net trade, shares of import growth to growth in IIT, shares of export growth to growth in IIT of commodity i between country j and country k. TT_ijk_, M_ijk_, X_ijk_, m_ijk_ and x_ijk_ are total trade of commodity i between countries j and k, imports of country j from country k of commodity i, exports of country j to country k of commodity i, growth rates during the period in M_ijk_ and X_ijk_, respectively.


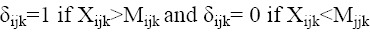


Then,


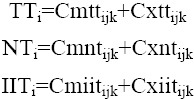


TT_i_, NT_i_ and IIT_i_ are respectively the percentage changes in total trade, net trade and IIT of commodity i during the period. These measures illuminate import and export patterns over time while the Grubel-Lloyd index disregards (Menon & Dixon, 1996).

The required data were obtained from Iran’s Ministry of Industry, Mine and Trade, Iran’s pharmaceutical Statistics, the Islamic Republic of Iran’s Customs Administration, and World Bank and International Trade Center. The data were processed at the 4-digit Harmonized System International Classification level during time period 2001-2012. The research population consisted of trade and economic data associated with trading partners of Iran in the pharmaceutical sector.

## 3. Results

The results are presented in two sections. In the first section, Iran’s comparative advantage in pharmaceuticals by the indicators of trade specialization, export propensity, import penetration, Balassa’s revealed comparative advantage, and Vollrath’s revealed competitive advantage measured and compared with other pharmaceutical exporting countries. In the second section, the extent and growth of Iran’s intra-industry trade in pharmaceuticals using the Grubel-Lloyd and Menon-Dixon indexes were calculated and evaluated.

### 3.1 Iran’s Comparative Advantage in Pharmaceuticals

The negative value of *trade specialization index* indicates that Iran is a net importer of pharmaceuticals. In spite of the trade imbalance of Iran’s pharmaceuticals, the value of TSI has increased during the study period (from -0.940 in 2001 to -0.844 in 2010), and shows the positive impact of trade policies of government on pharmaceutical export. Thus, Iran more engaged in intra-industry trade in pharmaceuticals.

**Table 1:** Trade Specialization Index for Iran’s pharmaceutical products, 2001-10

Year	Trade Specialization Index (TSI)
2001	-0.940
2006	-0.898
2010	-0.844

The trend in *export propensity* *index* of Iran’s pharmaceuticals is ascendant during 2001-10 (8.04-34.28), due to an increase in domestic pharmaceutical production in Iran. Then, the index declined in 2011 and 2012 because of economic sanctions and rapid growth in domestic sales of pharmaceuticals produced in Iran.

The *import penetration ratio* of Iran’s pharmaceuticals has decreased during the period 2001-9 (73.94-69.46), mostly as a result of the growth of total domestic consumption of pharmaceuticals in Iran. But the ratio increased sharply from 2010, that economic sanctions and consequently pharmaceutical industry’s dependence on primary material procurement for pharmaceutical manufacturing in Iran was one of the most significant factors which is positively affected this ratio.

Also, increasing price of primary material besides impossibility of subsidy exchange induce the producing companies pursue the buying policy of cheaper primary materials for profitability. As a result, quality of products falls too low. So the decline of quality of the domestic products is other important factors in increasing import penetration ratio.

Iran’s pharmaceutical *export/import ratio* has risen from 3% in 2001 to 11% in 2012, reflects a significant increase in pharmaceutical competitiveness of Iran. Increasing in Iran’s pharmaceutical competitiveness is due to the trade policies of government on pharmaceutical export.

As observed in Table 2, Iran’s *Revealed comparative advantage index* in pharmaceuticals is less than one, while the index for Switzerland, Belgium, United Kingdom, France, Germany, Italy and United States is greater than one. Particularly, Switzerland has the maximum index of revealed comparative advantage in pharmaceuticals which refers to its high export share of pharmaceuticals than the total world exports. The low value of Iran’s revealed comparative advantage index indicates the lower export share of pharmaceuticals than the total world exports, which proves that Iran has a revealed comparative disadvantage in pharmaceuticals.

**Table 2:** Revealed comparative advantage indexes of Iran and major pharmaceutical exporting countries, 2001-12

Country	RCA		

	2001	2006	2012
Belgium	2.432	4.24	4.35
France	2.175	2.073	2.706
Germany	1.527	1.584	2.077
Iran	0.026	0.027	0.032
Italy	1.36	1.289	1.73
Switzerland	6.94	7.701	10.429
U.K.	2.37	2.295	2.982
U.S.	0.895	1.016	1.124

According to results of Table 3, all three indexes of *RTA*, *REA* and *RC* are negative for Iran, so Iran has a competitive disadvantage in pharmaceutical products. Also, the negative values of RTA and RC indexes show that trade deficit exist in the industry. The negative value of REA indicates the low export share of Iran’s pharmaceutical than the total world exports.

**Table 3:** Vollrath’s indexes of revealed competitive advantage in pharmaceuticals for Iran and other exporting countries, 2001-12

Country	RTA			REA			RC		

	2001	2006	2012	2001	2006	2012	2001	2006	2012
Belgium	0.243	0.45	1.37	0.964	1.635	1.628	0.097	0.092	0.313
France	1.008	0.946	1.219	0.86	0.8	1.084	0.556	0.553	0.532
Germany	0.635	0.179	0.693	0.488	0.533	0.837	0.494	0.111	0.357
Iran	-1.271	-0.748	-0.573	-3.672	-3.631	-3.485	-3.931	-3.375	-2.98
Italy	0.011	0.058	-0.048	0.329	0.27	0.583	0.008	0.045	-0.026
Switzerland	4.312	5.614	10.085	2.144	2.308	2.705	0.703	0.818	1.121
U.K.	1.175	1.341	1.578	0.955	0.913	1.188	0.602	0.772	0.656
U.S.	0.193	0.119	-0.049	-0.129	0.016	0.128	0.248	0.124	-0.042

It is clear from analyses of mentioned indexes that between the pharmaceutical exporting countries, Iran has a high degree of comparative disadvantage in pharmaceuticals.

### 3.2 Iran’s Intra-Industry Trade in Pharmaceuticals

This section provides an analysis of the extent and growth of Intra-Industry Trade in Iran’s pharmaceuticals. Economies of scale for Iran’s pharmaceutical products have increased from 10 in 2001 to 16.2 in 2010, indicates a rapid growth in volume of Iran’s pharmaceutical transactions by reason of cost advantages in their productions. Also, the degree of product differentiation has increased from 0.058 in 2001 to 0.095 in 2010. Therefore, increasing economies of scale and product differentiation have caused the growth of Iran’s intra-industry trade in pharmaceuticals.

#### 3.2.1 Extent of Intra-Industry Trade in Pharmaceuticals

The standard index of Grubel-Lloyd (GL_i_) is applied to calculate the extent of intra-industry trade of Iran and major pharmaceutical exporting countries with the rest of the world and as well bilateral intra-industry trade among Iran and 18 of its trading partners.

Based on results of table 4, the extent IIT for following countries except Iran were high. In other words, these countries were almost net exporters of pharmaceuticals.

**Table 4:** Grubel-Lloyd index of intra-industry trade in pharmaceuticals for Iran and major exporting countries, 2001-12

Country	2001	2006	2012	Net exporter or importer
Belgium	91.18	94.95	87.37	Net exporter
France	74.96	80.86	86.31	Net exporter
Germany	69.92	86.6	77.7	Net exporter
Iran	5.97	10.22	19.85	Net importer
Italy	96.42	98.64	98.71	Net exporter
Switzerland	70.97	66.5	54.56	Net exporter
U.K.	82.49	81.31	89.62	Net exporter
U.S.	87.61	74.42	76.71	Net exporter

Specifically, the extent of Iran’s Intra-industry trade in pharmaceuticals has increased from about 6% in 2001 to about 20% in 2012. The increase in the extent of IIT in Iran’s pharmaceuticals was probably a result of the improving product differentiation and increasing in the number of produced pharmaceuticals over the period.

High income, trade liberalization, customs unions, common borders and large economies of scale in pharmaceutical production seems to be caused a higher IIT for industrial countries. Low levels of Iran’s Intra-industry trade indicates that pharmaceutical trade in this country is derived from trade complementary that accentuates the benefits of *inter-industry trade*. Thus, Iran is about a net importer of pharmaceuticals.

Table 5 shows that most indexes of bilateral intra-industry trade among Iran and its trading partners are very low. The pharmaceutical import values of Iran from net exporters are noticeably high, compared with pharmaceutical exports from Iran to these countries. Furthermore, more than a half of Iran’s pharmaceutical exports are supplied to the neighboring countries such as Afghanistan, Iraq and etc. The pharmaceutical import values from these countries to Iran than the values of pharmaceutical exports from Iran to these countries are small. Consequently, intra-industry trade indexes among Iran and these countries for pharmaceutical products are low. On the other hand, Iran’s bilateral intra-industry trade index for some of its trading partners, such as Tajikistan and Azerbaijan are somewhat high in some years, but have fluctuated during the period, which refers to the values of exports and imports in that years.

**Table 5:** Bilateral indexes of intra-industry trade in pharmaceuticals between Iran and 18 major trading partners, 2001-12

Country	2001	2006	2012
Afghanistan	0.056	0.086	1.987
Armenia	24.489	27.635	3.599
Azerbaijan	4.181	6.395	39.046
Belgium	0.009	0.996	0.208
China	0.184	3.538	3.153
France	0.044	9.727	0.327
Germany	0.745	7.024	3.548
India	1.498	10.533	6.173
Iraq	4.006	1.887	3.497
Jordan	20.808	6.192	2.021
Pakistan	29.03	28.226	2.055
Somalia	0.499	0.801	0.16
Sudan	0.395	0.349	0.178
Switzerland	0.014	14.924	0.538
Tajikistan	2.715	54.826	49.845
Ukraine	75.389	28.64	10.999
United Kingdom	1.207	6.555	5.741
Yemen	6.694	7.728	10.614

#### 3.2.2 Growth in Intra-Industry trade in Pharmaceuticals

In this section, the developed indexes of Menon-Dixon used which express changes in intra-industry trade in terms of the shares of exports and imports to growth in total trade, net trade and intra-industry trade.

The results presented in Table 6 show that the shares of export growth to total trade (Cxtt) is lower than those of import growth to total trade (Cmtt), indicates the rapid growth in pharmaceutical imports by Iran in that period. The percentage growth in Iran’s total pharmaceutical trade (TT_i_) has increased during 2001-8, but has dropped in the period 2009-12 for many reasons such as economic sanctions of the country.

**Table 6:** Percentage growth in total trade (TT_i_), net trade (NT_i_) and intra-industry trade (IIT_i_) for Iran’s pharmaceuticals, 2001-12

Index/Year	2001-4	2005-8	2009-12
Cxtt	2.13	0.77	1.81
Cmtt	10.33	13.86	7.86
TT_i_	12.46	14.64	9.67
Cxnt	-2.37	-0.87	-2.13
Cmnt	11.45	15.53	8.92
NT_i_	9.08	14.66	6.79
Cxiit	43.70	13.92	23.74
Cmiit	0.00	0.00	0.00
IIT_i_	43.70	13.92	23.74

The shares of export growth to the growth in net trade (Cxnt) are all negative in the study periods since imports are the dominant factor of net trade in Iran’s pharmaceutical industry. The shares of import growth to the growth in intra-industry trade (Cmiit) are all zero. The shares of export growth to the growth in intra-industry trade (Cxiit) have fluctuated during the study periods and have decreased from %43.7 in the first period to %23.74 in the third period. So, all of the growth in Iran’s intra-industry trade in pharmaceuticals is mostly because of export growth than import growth.

Table 7 shows the increase in the percentage growth in total trade between Iran and its trading partners are due to the growing in the shares of exports and imports to total trade with these countries. In contrast, the rise in pharmaceutical trade between Iran and major pharmaceutical exporting countries such as Switzerland, Belgium, France, Germany and United Kingdom arises from the increasing in the shares of import growth to growth in total trade (Cmtt) than those of export growth (Cxtt).

**Table 7:** Percentage growth in total trade (TT_i_) for Iran’s bilateral trade in pharmaceuticals, 2001-12

Country	2001-4			2005-8			2009-12		

	Cxtt	Cmtt	TT_i_	Cxtt	Cmtt	TT_i_	Cxtt	Cmtt	TT_i_
Afghanistan	45.72	0.61	46.34	9.22	0.54	9.77	16.6	30.57	47.17
Armenia	-11.83	6.21	-5.62	22.3	-1.32	20.98	157.42	6.83	164.26
Azerbaijan	627.63	0.23	627.86	-20.06	4.95	-15.11	14.98	13.44	28.43
Belgium	0.02	-14.36	-14.34	0.19	2.25	2.44	0.08	100.15	100.23
China	0.59	17.19	17.78	0.35	19.94	20.29	6.08	62.3	68.39
France	0.05	-5.37	-5.32	0.38	-7.57	-7.18	0.37	550.77	551.14
Germany	0.44	-1.82	-1.38	-0.04	50.24	50.2	3.7	109.93	113.63
India	1.03	-16.65	-15.62	0.16	5.6	5.77	42.31	126.27	168.58
Iraq	30.37	79.68	110.05	3.01	9.87	12.88	20.92	217.69	238.61
Jordan	34.68	1.84	36.51	67.25	0.2	67.45	19.15	0.12	19.27
Pakistan	148.57	1.59	150.16	-5.67	5.1	-0.57	469.7	22.54	492.24
Somalia	54.55	0.18	54.73	47.95	0.08	48.03	32.02	0.01	32.03
Sudan	55.36	0.07	55.44	19.8	0.04	19.84	43.75	0.03	43.78
Switzerland	0.2	1.14	1.34	1.47	29.44	30.92	0.18	148	148.18
Tajikistan	14.6	45.86	60.46	10.03	6.42	16.45	51.84	6.48	58.32
Ukraine	29.73	-4.73	25	22.29	0.42	22.72	24.59	0.37	24.97
United Kingdom	0.03	-22.63	-22.6	0.1	-12.1	-11.99	0.5	96.14	96.64
Yemen	48.36	2.32	50.68	136.28	7.66	143.94	14.52	1.23	15.75

Trade policies of government about pharmaceutical exports and also the reduction of tariff rate have resulted in the growth in Iran’s pharmaceutical trade with its trading partners, particularly in the period 2009-12.

According to results of table 8, the shares of import growth to growth in net trade (Cmnt) in pharmaceuticals between Iran and its neighboring countries such as Afghanistan, Iraq, Tajikistan, Yemen and etc are all negative, implying that the shares of exports exceed imports from these countries during the three study periods. Though, the growth in net trade (NT_i_) with some of the neighboring countries has declined over time.

**Table 8:** Percentage growth in net trade (NTi) for Iran’s bilateral trade in pharmaceuticals, 2001-12

Country	2001-4			2005-8			2009-12		

	Cxnt	Cmnt	NT_i_	Cxnt	Cmnt	NT_i_	Cxnt	Cmnt	NT_i_
Afghanistan	45.79	-0.62	45.18	9.26	-0.55	8.71	16.44	-31.19	-14.75
Armenia	-28.36	-12.18	-40.54	30.31	2.06	32.37	183.81	-9.23	174.58
Azerbaijan	629.59	-0.23	629.36	-22.15	-6.16	-28.31	10.86	-19.12	-8.26
Belgium	-0.02	-14.38	-14.4	-0.2	2.26	2.07	-0.08	100.44	100.36
China	-0.6	17.03	16.43	-0.36	20.28	19.92	-7.02	65.16	58.14
France	-0.05	-5.48	-5.53	-0.42	-8.39	-8.81	-0.38	553.24	552.86
Germany	-0.44	-1.94	-2.38	0.03	49.69	49.72	-3.82	112.11	108.29
India	-1.08	-17.51	-18.59	-0.18	5.53	5.35	-61.13	129.98	68.86
Iraq	41.32	-121.51	-80.19	3.63	-10.58	-6.95	21.36	-230.21	-208.85
Jordan	38.57	-2.11	36.46	70.23	-0.23	70	19.53	-0.12	19.41
Pakistan	158.31	-1.82	156.49	-9.25	-6.78	-16.03	476.79	-23.02	453.77
Somalia	54.76	-0.18	54.58	48.1	-0.08	48.02	32.07	-0.01	32.06
Sudan	55.55	-0.07	55.48	19.84	-0.04	19.8	43.86	-0.03	43.83
Switzerland	-0.2	1.06	0.86	-1.67	30.65	28.98	-0.19	149.82	149.63
Tajikistan	20.01	-61.3	-41.3	22.79	-14.46	8.34	64	-12.24	51.76
Ukraine	42.07	11.86	53.93	29.15	-0.57	28.58	27.25	-0.38	26.86
United Kingdom	-0.03	-23.89	-23.93	-0.12	-16.22	-16.34	-0.55	101.26	100.71
Yemen	52.43	-2.52	49.91	147.22	-9.4	137.82	15.03	-1.49	13.55

In contrast, the growth in net trade (NT_i_) between Iran and major pharmaceutical exporting countries such as Switzerland, Belgium, France, Germany and United Kingdom has increased during the period 2009-12.

These results demonstrate the shares of export growth to growth in net trade (Cxnt) between Iran and pharmaceutical exporting countries are negative, means that shares of imports exceed exports.

Table 9 shows all of the growth in intra-industry trade with neighboring countries such as Afghanistan, Iraq, Tajikistan and Yemen is because of the share of import growth to growth in intra industry trade (Cmiit) and the share of export growth (Cxiit) by these countries are zero. As well, the growth in intra-industry trade between Iran and Switzerland, Belgium, France, Germany and United Kingdom is due to the share of exports to growth in intra-industry trade (Cxiit). The shares of imports (Cmiit) for these countries are all zero, reflects that Iran is a net importer of pharmaceuticals from these countries.

**Table 9:** Percentage growth in intra-industry trade (IIT_i_) for Iran’s bilateral trade in pharmaceuticals, 2001-12

Country	2001-4			2005-8			2009-12		

	Cxiit	Cmiit	IIT_i_	Cxiit	Cmiit	IIT_i_	Cxiit	Cmiit	IIT_i_
Afghanistan	0	386.18	386.18	0	186.2	186.2	0	3,076.46	3,076.46
Armenia	0	24.7	24.7	0	-10.77	-10.77	0	45.67	45.67
Azerbaijan	0	67.73	67.73	0	62.16	62.16	0	91.48	91.48
Belgium	66.27	0	66.27	35.3	0	35.3	15.05	0	15.05
China	121.65	0	121.65	21.41	0	21.41	94.49	0	94.49
France	81.76	0	81.76	11.45	0	11.45	40.03	0	40.03
Germany	58.22	0	58.22	-29.28	0	-29.28	294.88	0	294.88
India	38.33	0	38.33	4.56	0	4.56	272.37	0	272.37
Iraq	0	452.85	452.85	0	264.73	264.73	0	7,981.55	7,981.55
Jordan	0	22.41	22.41	0	3.02	3.02	0	11.69	11.69
Pakistan	0	28.91	28.91	0	28.57	28.57	0	2,169.76	2,169.76
Somalia	0	66.77	66.77	0	17.09	17.09	0	14.75	14.75
Sudan	0	41.02	41.02	0	17.21	17.21	0	22.94	22.94
Switzerland	355.66	0	355.66	30.81	0	30.81	16.31	0	16.31
Tajikistan	0	396.48	396.48	0	23.19	23.19	0	30.65	30.65
Ukraine	8.46	-12.48	-4.02	0	-0.89	-0.89	0	7.45	7.45
United Kingdom	2.59	0	2.59	1.94	0	1.94	15.1	0	15.1
Yemen	0	56.99	56.99	0	94.97	94.97	0	15.45	15.45

## 4. Discussion

Pharmaceutical products have been classified as a technology-intensive industry. Despite the growth of pharmaceutical exports due to trade policies of government, the value of Iran’s pharmaceutical imports has been more than its exports over the past decade. The major export destinations for Iran’s pharmaceuticals are neighboring countries, Southeast Asian countries (CIS) and some African countries. Most of the Iran’s pharmaceutical imports are in the form of primary materials and finished products from Europe and some Asian countries such as India and China; reveals that foreign dependence of Iran for pharmaceutical producing.

The results of trade specialization index, export propensity, import penetration and export/import ratio show that Iran is a net importer of pharmaceuticals. The trade specialization index of Iran’s pharmaceutical industry is negative during the study period. However, the growth rate of this index has raised due to government’s trade policies. The export propensity and import penetration have somewhat increased and declined over the years 2000 to 2009, respectively. These are due to an increase in domestic production of pharmaceuticals and its rising consumption in Iran during the same period. But economic sanctions and consequently strong dependence of country’s pharmaceutical industry for supply of primary materials has been conversely affected the both indexes mentioned above from 2010 onwards. In terms of competitiveness, the export/import ratio of Iran’s pharmaceuticals has increased over the under review period, demonstrating the positive impact of Iran’s trade and industrial policies on pharmaceutical exports.

The analysis of Balassa’s RCA index displayed that, among major pharmaceutical exporting countries, Iran has a high degree of comparative disadvantage in pharmaceuticals. Iran’s RCA index is less than one for pharmaceutical products, reflects Iran has a small export share of pharmaceuticals than the total world exports. However, Iran’s RCA index represents a progressive trend in pharmaceutical industry, which can increase the share of pharmaceutical in the export basket of non-oil commodities with investment in this industry. Many factors such as fluctuation in primary material price and access to them, technological progresses etc. have significant impacts on this industry.

Also, the negative values of Vollrath’s indexes of revealed competitive advantage reflect that Iran has a competitive disadvantage in pharmaceuticals because of a lower export share of pharmaceuticals than the total world exports. Hence, based on the results of Balassa’s revealed comparative advantage and Vollrath’s revealed competitive advantage indexes, it is concluded that Iran has a comparative disadvantage in pharmaceuticals.

There is intra-industry trade in pharmaceutical industry due to Iran exports some pharmaceutical products, whereas imports some other types of them at the same time. Iran’s bilateral trade in pharmaceuticals cannot be justified by the theory of comparative advantage because of intra-industry trade resulting from economies of scale, product differentiation under imperfect competition.

The analysis of Grubel-Lloyd index indicates that, among the major pharmaceutical exporting countries, Iran has a small intra-industry trade in pharmaceuticals. Specifically, the extent of intra-industry trade of these products is estimated about 6%-20% during 2001 to 2012. However, Iran’s intra-industry trade in pharmaceutical products shows an improvement during the study period. Increasing the growth rate of exports due to an improvement in product differentiation and an increase in the number of pharmaceutical’s domestic production are caused a rise in the extent of Iran’s intra-industry trade in pharmaceuticals during the period.

The extent of bilateral intra-industry trade in pharmaceuticals among Iran and its major trading partners are low due to the values of pharmaceutical imports from these countries to Iran are very small rather than the values of pharmaceutical exports from Iran to these countries. The low extent of bilateral intra-industry trade between Iran and its trading partners in pharmaceuticals shows the trading model of Iran’s pharmaceutical industry is most inter-industry trade rather than intra-industry trade.

Based on the results of Menon-Dixon indexes, the growth in Iran’s total trade and net trade in pharmaceuticals is due to the increase in pharmaceutical imports during the study period. Also, the growth of Iran’s intra-industry trade in pharmaceuticals is as a result of the increase in the shares of pharmaceutical exports than imports. In terms of bilateral trade, the growth in total trade and net trade in pharmaceuticals between Iran and its trading partners are due to a rise in the shares of import growth from major pharmaceutical exporting countries to Iran and the shares of export growth from Iran to its neighboring countries. Similarly, the growth in intra-industry trade in Iran’s pharmaceuticals is because of the shares of Iran’s imports from major pharmaceutical exporting countries and the shares of exports to neighboring countries of Iran.

So far, a comprehensive study has not been done in the field of Iran’s international trade in pharmaceuticals. Karn assessed the patterns and factors influencing Australia’s international trade in pharmaceutical industry. The result of our research was mostly alike to Karn’s study. According to his results, from among the industrial countries, Australia has a high degree of comparative disadvantage in pharmaceutical sector due to its small share in the world’s total exports of pharmaceuticals. Moreover, Australia has a low extent of intra-industry trade of pharmaceutical products compared to other countries that are considered as the major exporters of drugs (Chuankamnerdkarn, 1997).

## 5. Conclusions

There is a significant potential for Iran in expanding its IIT in pharmaceutical industry because of the small extent of intra-industry trade of these products. So, large adjustment costs for liberalization of pharmaceutical trade, low competitiveness and globalization rates are the results of small extent of Iran’s intra-industry trade in pharmaceutical products. Hence, to facilitate integration into the global economy, Iran should consider developing IIT in pharmaceuticals as well as taking care of existing comparative advantages. In this regard, the most important policy recommendations are as follows:

1). Paying attention to intra-industry trade in pharmaceutical sector can help promote pharmaceutical exports and, consequently, non-oil exports. In this regard, it is recommended that Iran pays more attention to the factors influencing intra-industry trade of pharmaceutical products (such as product differentiation, economies of scale, and consumers’ preferences at the international level) besides protecting the common advantages.
2). The government should grant certain permits for importing specific drugs so that they can be used by researchers who are trying to manufacture their domestic equivalents. This will facilitate the transfer of technology and technical knowledge and reduces Iran’s dependence in this regard.
3). Certain strategies should be formulated in order to improve the competitiveness of domestic pharmaceutical companies and, consequently, further prepare Iran in the ongoing process to join WTO.
4). Promotion of marketing and sales through earning international certificates (GMP and DMF) is necessary as these achievements introduce pharmaceutical products to the regional and global markets and, consequently, lead to an increase in exports.
5). Exports of pharmaceutical products should be viewed as a principle and considered in all future development plans as the essence so that the country can take advantage of the new opportunities in the international trade market by relying on its comparative advantages.
6). The pricing methods should be corrected in order to provide a competitive conditions in Iran’s pharmaceutical markets and to make it possible to manufacture higher quality pharmaceutical products.
7). Import of drugs that have similar domestic counterpart should be banned or restricted.

Considering the above-mentioned factors, paying attention to comparative advantage and intra-industry trade could play a valuable role in helping pharmaceutical companies and policy makers in their efforts to promote pharmaceutical trade.

## Ethical Issues

Ethical issues have been exactly considered by the authors.
